# Therapeutic Potential of HUMSCs in Female Reproductive Aging

**DOI:** 10.3389/fcell.2021.650003

**Published:** 2021-05-10

**Authors:** Qiaojuan Mei, Hongbei Mou, Xuemei Liu, Wenpei Xiang

**Affiliations:** ^1^Institute of Reproductive Health and Center for Reproductive Medicine, Tongji Medical College, Huazhong University of Science and Technology, Wuhan, China; ^2^Reproductive Medicine Centre, Yantai Yuhuangding Hospital of Qingdao University, Shandong, China

**Keywords:** reproduction, aging, mesenchymal stem cells, premature ovarian failure, transplantation

## Abstract

With the development of regenerative medicine, stem cells are being considered more frequently for the treatment of reproductive aging. Human umbilical cord mesenchymal stem cells have been reported to improve the reserve function of aging ovaries through their homing and paracrine effects. In this process, paracrine factors secreted by stem cells play an important role in ovarian recovery. Although the transplantation of human umbilical cord mesenchymal stem cells to improve ovarian function has been studied with great success in animal models of reproductive aging, their application in clinical research and therapy is still relatively rare. Therefore, this paper reviews the role of human umbilical cord mesenchymal stem cells in the treatment of reproductive aging and their related mechanisms, and it does so in order to provide a theoretical basis for further research and clinical treatment.

## Introduction

**FIGURE 1:**
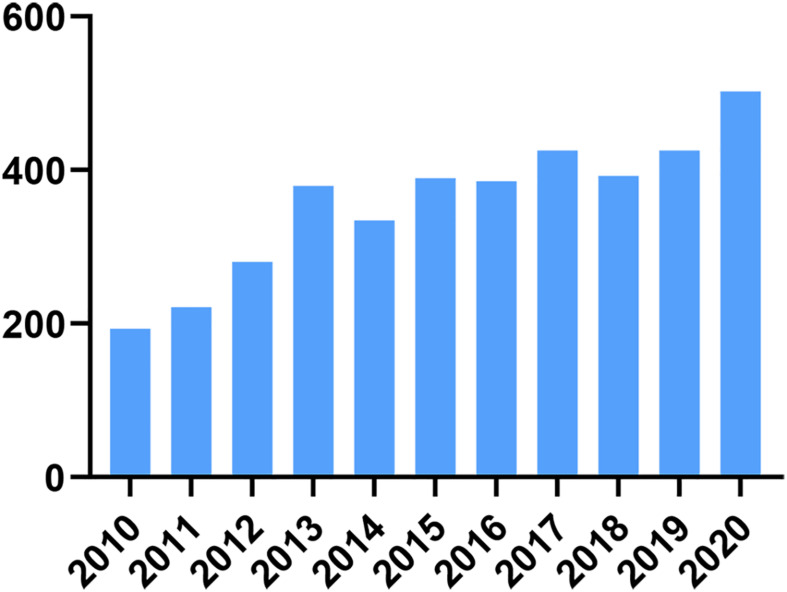
Number of papers published in HUMSCs research in the last decade.

Delaying childbearing is an important social change that has led to an increasing number of women wishing to slow the rate of reproductive aging. It is important to note that female reproductive aging varies widely among individuals, and nearly 20% of infertility counselors have symptoms of premature ovarian failure (POF) (May-Panloup et al., 2016). Currently, hormone replacement therapy is the main treatment for delaying ovarian aging; however, hormone replacement therapy increases the risk of breast, endometrial, and ovarian cancers (Rees, 2011). Other methods such as freezing ovarian tissue, gametes, and embryos, although diversifying the options for women to preserve their fertility, patients are limited by the corresponding treatment criteria and cannot be administered on a large scale (Donnez and Dolmans, 2018). In the last decade, with the development of regenerative medicine, research on human umbilical cord mesenchymal stem cells (HUMSCs) has increased year by year, which proves that researchers have increasingly understood the function and potential of human umbilical cord MSCs, as seen in Figure 1. In the field of female reproduction, numerous studies (Zhao et al., 2019) have confirmed the recovery potential of HUMSC transplantation/infusion in animal models of POI. In these studies, researchers first tested them in rodent models that were induced by chemotherapeutic agents such as cyclophosphamide (Jalalie et al., 2021), thus mimicking the therapeutic effects of chemotherapy in women. Natural aging (Yang et al., 2020) and knockout (Persani et al., 2010; Chen et al., 2020) models can also be used, the latter being particularly useful to study known or suggested genetic causes of POI. In these models, the therapeutic effects of HUMSCs are assessed by a range of aspects of ovarian function, including follicular development, granulosa cell apoptosis, neoangiogenesis, serum hormone levels and, of course, most importantly, pregnancy rates.

Despite the overwhelmingly positive evidence obtained by exploring the repair potential of HUMSCs in animal POI models, little has been published in terms of human studies and robust clinical trials. In fact, much of the work in this area is pre-emptive, observational and uncontrolled; thus, many of these studies need to be interpreted with extra caution due to the established potential for unintended ovulation and pregnancy. In one such study (Yan et al., 2020), researchers transplanted HUMSCs by *in situ* injection under vaginal ultrasound guidance into the ovaries of 61 patients with primary ovarian insufficiency and subsequently monitored for side effects, vital signs and clinical changes, and collected hematological and imaging parameters during follow-up. The results observed normal clinical behavior in all patients without serious side effects and treatment-related complications; after stem cell treatment, POI patients with shorter amenorrhea appeared to be more likely to obtain mature follicles, while patients with better ovarian status were more likely to have better outcomes with HUMSCs injection, and 4 POI patients had successful clinical deliveries after HUMSCs transplantation. This suggests that clinical transplantation of HUMSCs rescued ovarian function in patients with POI, as evidenced by increased follicular development and improved egg collection. However, due to the limitations of HUMSCs transplantation and the lack of reports from clinically relevant studies, this method has not been widely used in the clinical treatment of female reproductive aging patients, and its clinical efficacy and safety still need further study.

## Methods

**FIGURE 2:**
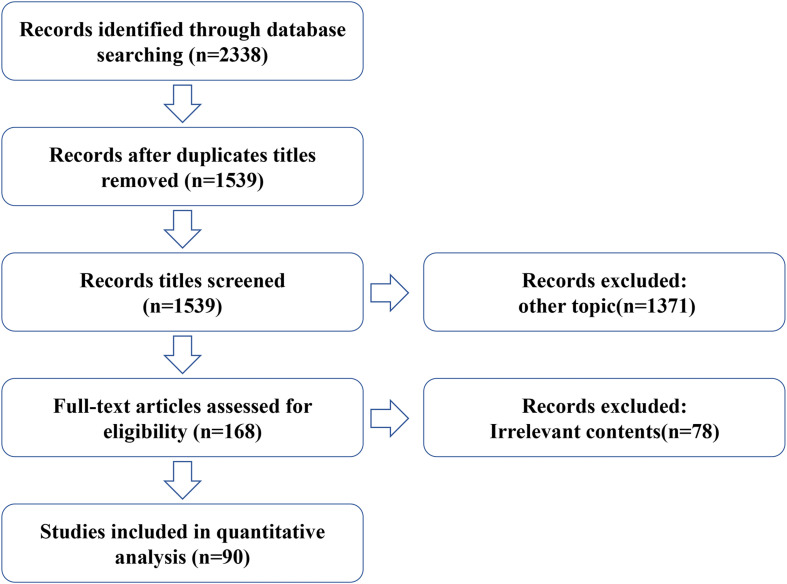
Schematic of study selection.

For this review, we conducted a systematic online literature search of PubMed and Web of Science databases using a total of three strategies including literature search, study selection, and summary of results, and searched all published articles since the creation of the databases up to 2021. We used the following queries (“Umbilical Cord Mesenchymal Stem Cell” or “Human umbilical cord mesenchymal stem cell”) and (“ovary aging” or “female reproductive aging” or “POF” or “POI”). Both animal and human studies were considered appropriate for this review. In addition, all relevant studies were identified and included. Any duplicate articles were excluded. After screening the titles and/or abstracts, articles were excluded if they were found to be irrelevant to the study. A total of 2,338 records were retrieved from both databases. After excluding duplicate titles and other subject articles, the full text of 188 articles was reviewed and 90 were considered relevant and included in this review (Figure 2).

## Manifestations of Female Reproductive Aging

Reproductive aging is a natural process that occurs in all women, eventually leading to reproductive senescence and menopause. Reproductive aging is considered to be the result of a progressive decline in the number and quality of oocytes in the follicles present in the ovarian cortex (Broekmans et al., 2009); menopause is the permanent termination of menstruation due to the loss of ovarian follicular activity. Perimenopause is the onset of menopause, which is characterized by menstrual irregularities and vasodilatory symptoms, and ends within 12 months after menopause (Rees, 2011). Most women typically experience menopause between the ages of 45 and 55 years (Brotherston, 2015). Perimenopause can last for years or even decades and brings with it many menopausal symptoms such as hot flashes, vaginal atrophy, osteoporosis, and depression (Ortmann et al., 2011; Weber et al., 2014). Delayed childbearing has led to an increasing number of women wishing to undergo menopause later, and an improved quality of life means that women wish to avoid the distress of menopausal symptoms and slow down the rate of ovarian aging. In addition, the number of follicles is limited. The ovary establishes millions of non-growing follicles (NGF) at approximately 5 gestational ages, declines to approximately 1,000 at the onset of menopause, and is depleted through follicular atresia and apoptosis 12–14 years after menopause (Richardson et al., 1987; Faddy et al., 1992). Therefore, the fundamental question of how to fully utilize NGF to delay ovarian aging and treat perimenopausal syndrome is a serious issue in today’s society.

## Characteristics of Human Umbilical Cord Mesenchymal Stem Cells

HUMSCs were obtained from human umbilical cord Wharton’s colloids. Although mesenchymal stem cells (MSCs) from different sources share many similar characteristics, they also exhibit individual properties. HUMSCs are biologically superior to MSCs isolated from amniotic membrane (A-MSC), chorionic villus (C-MSC), and placental metaplasia (D-MSC) in that HUMSCs secrete higher levels of paracrine factors (Araújo et al., 2017; Wu et al., 2018; Guan et al., 2019) and have a greater proliferative capacity and weaker immunosuppressive function than MSCs from the metaplastic basement membrane in the placental matrix (Chen et al., 2015). Among them, the ability of HUMSCs to modulate immune responses makes them an important source of stem cells for allogeneic transplantation therapy without immune rejection (Fong et al., 2011), and studies have shown that undifferentiated HUMSCs have no evidence of direct immune rejection *in vivo*, can survive longer after transplantation in ovarian tissue (Song et al., 2016), and are more acceptable in allogeneic transplantation (Seshareddy et al., 2008). Numerous studies have demonstrated the low immunogenicity of human umbilical cord MSCs, whose transplantation can rescue organ damage but not cause immune rejection (Wang et al., 2013; Song et al., 2016). In addition, HUMSCs can be obtained from discarded umbilical cords with few ethical issues (Bongso and Fong, 2013), and their paracrine function can also participate in the repair of tissue damage by enhancing endogenous cell function. According to the available studies, HUMSCs have achieved some good results in improving ovarian reserve function in reproductive aging.

## Transplantation Method of Human Umbilical Cord Mesenchymal Stem Cells

The results achieved by transplantation of HUMSCs in animal models of POI have been well established in previous studies, but few studies have investigated the optimal transplantation method by which HUMSCs can delay ovarian aging. The key issue in the application of stem cells in practice is to adjust the dose and method of administration to optimize the results. Although various debates still exist, all predictable outcomes are very promising. Among researchers, transplantation methods vary, with existing studies mostly using *in situ* ovarian microinjection (MI) (Fu et al., 2008) and intravenous transplantation (IV) (Wang et al., 2013). Zhu et al. (2015) applied membrane-labeled HUMSCs via *in situ* ovarian microinjection and tail vein injection directly to a rat model of POF, and found that HUMSCs via MI were distributed only in the ovaries and uterus. While HUMSCs via IV were detected in ovaries, uterus, kidneys, liver and lungs, both methods yielded similar results in later observations, despite the fact that ovarian injection restored ovarian function faster (Zhu et al., 2015). Considering the two ways of transplanting HUMSCs, IV represents a more minimally invasive approach that causes less damage than invasive MI, involves a shorter recovery time, and provides an opportunity to deliver doses of HUMSCs at different concentrations. However, tail vein injections carry a risk of thrombosis due to larger or insufficiently dissociated cells. How to judge the optimal transplantation method still needs further research and discussion. In addition to the transplantation of HUMSCs alone, the rapid development of tissue engineering has given us a blueprint for new transplantation methods. For example, in a rat uterine scar model, the application of a scaffold/HUMSCs system both promotes collagen degradation in the uterine scar through the paracrine effect of stem cells and also allows for longer local retention of HUMSCs with longer duration of action (Xu et al., 2017). Recently, Wang et al. applied the HUMSCs/HA-GEL system for dual repair of endometrial damage and adhesions in a non-human primate model of uterine adhesions, with the intervention of new materials allowing the maximum effect of HUMSCs (Wang et al., 2020a). Considering these existing studies, a new breakthrough in intra-ovarian cell transplantation therapy may be possible through the exploration of combined vector transplantation.

## The Role of Human Umbilical Cord Mesenchymal Stem Cells in the Treatment of Ovarian Aging

### Migration and Homing of Human Umbilical Cord Mesenchymal Stem Cells

Simply put, stem cells homing, means that they can migrate directly and impulsively into damaged tissues and survive stimulation by multiple factors to promote ovarian recovery. Studies have shown that unstimulated HUMSCs express a wide range of mRNAs encoding cytokines, chemokines and their receptors. Among them CXCL12/stromal cell-derived factor 1 alpha (SDF-1 alpha), CX3CL1/fractalkine and CXCL10/IFN-γ (IFN-γ inducible protein (IP-10) can lead to significant HUMSC migration (Croitoru-Lamoury et al., 2007). In the POF mouse model, trans-tail vein transplanted HUMSCs were observed to be unevenly distributed in the ovary. In the ovarian medulla, the number of HUMSCs was greater than that of the ovarian cortex and germinal epithelium, and the average number was significantly higher in the cortical region than in the germinal epithelium. The migration of HUMSCs to the medulla may be due to the fact that it consists mainly of stromal tissue rather than cortical and germinal epithelium, and that the medulla is rich in blood vessels, and the stromal region of ovarian tissue is a source of SDF-1 during injury and inflammation, which increases the migration of HUMSCs to this region (Liu et al., 2014). Also, physical barriers in ovarian tissue may be a limiting factor for the homing of HUMSCs in specific regions of the tissue. For example, the low number of MSCs migrating in the ovarian germinal epithelium may be related to the preventive effect of physical barriers such as the basement membrane and cell-cell attachment complexes (Auersperg et al., 2001). In contrast, HUMSCs were not observed in follicles, oocytes or oocytes, probably because tight junctions between follicular cells prevented the cells from entering the follicles. In addition, intracellular junctions and interstitial junctions of the inner and outer follicular membrane cells of mature follicles may also be a barrier to homing. Similarly, the barrier that exists around the follicle like the basement membrane could prevent HUMSCs from entering the follicle (Chang et al., 2016). The application of this biological property of stem cell homing to the management of cytokines during stem cell therapy may be important in the process of stem cell directed migration and could play a pioneering role in the study of cell therapy homing to precise foci.

### Paracrine Effects of Human Umbilical Cord Mesenchymal Stem Cells

**FIGURE 3:**
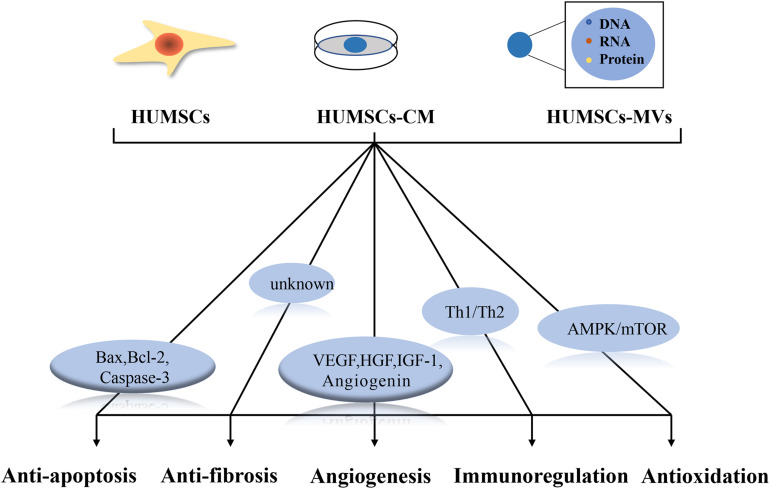
Possible mechanisms underlying the paracrine effect of human umbilical cord mesenchymal stem cells (HUMSCs). HUMSCs, HUMSCs-CM and HUMSCs-MVs all ameliorate ovarian senescence through paracrine effects. Regulation of Bax, Bcl-2, caspase-3 helps to inhibit apoptosis; VEGF, HGF, IGF-1 and angiopoietin play important roles in angiogenesis; Th1/Th2 cytokines play important roles in balanced local immune response; AMPK/mTOR signaling pathway plays an important role in antioxidant process. The mechanism of antifibrosis is not clear.

The secretion of chemokines, growth factors, and hormones by HUMSCs to affect adjacent cells is known as the paracrine effect. Paracrine secretion of HUMSCs plays an important role in angiogenesis, anti-inflammation, immunomodulation, anti-apoptosis, and anti-fibrosis, thus improving the microenvironment and promoting the recovery of injured tissues. Considering the paracrine effects of HUMSCs, some studies have also used human umbilical cord MSC-derived conditioned media (HUMSCs-CM) and human umbilical cord MSC microvesicles (HUMSCs-MVs), rather than HUMSCs themselves, to examine the therapeutic effects on damaged ovaries. Figure 3 summarizes the possible mechanisms of the paracrine effect of HUMSCs in female reproductive aging.

In a perimenopausal rat model, HUMSCs transplanted through the tail vein can increase estradiol (E2) and anti-Müllerian hormone (AMH) in the ovaries through up to 28 days of paracrine action—by secreting hepatocyte growth factor (HGF), Vascular endothelial growth factor (VEGF), and insulin-like growth factor 1 (IGF-1), improving ovarian architecture and increasing follicles (Li et al., 2017); In clinical trials, *in vitro*, umbilical cord mesenchymal stem cells (collagen/HUMSCs) on collagen scaffolds activated primordial follicles by phosphorylating FOXO3a and FOXO1; *in vivo*. Transplantation of collagen/HUMSC s into the ovaries of POF patients may improve overall ovarian function by increasing estradiol concentrations, improving follicular development and increasing the number of sinus follicles (Ding et al., 2018). Considering that there are no obvious conclusions regarding the long-term safety of HUMSCs *in vivo* (Wu et al., 2017), the use of HUMSCs-CM and HUMSCs-MVs is currently the preferred study by investigators.

Compared with normal medium, HUMSCs-CM protected both frozen and thawed ovarian tissues (Jia et al., 2017), and in a model of cisplatin-induced ovarian injury, HUMSCs-CM alleviated cisplatin-induced follicular failure and maintained fertility (Hong et al., 2020), and in follicular development, HUMSCs-CM improved the maturation and developmental potential of artificially activated the maturation and developmental potential of immature human oocytes after the activation (Akbari et al., 2017). Thus suggesting that perhaps conditioned medium from HUMSCs *in vitro*, induced by the same factors *in vivo*, could also be therapeutic for the disease. Consequently, conditioned medium may be an effective therapy applied in the clinic, and even that artificial cytokines could be a reality someday. However, there is still a need for relevant studies on the effect of conditioned medium for ovary aging.

Microvesicles (MVs), small vesicles with a diameter of <1 μm carrying membrane and cytoplasmic constituents of cellular origin, can transfer proteins, mRNA, and bioactive lipids to target cells through surface-expressed ligands and surface receptors, which affect the phenotype and function of the target cells (György et al., 2011; Bidarimath et al., 2017). It has been reported that MVs released from mesenchymal stem cells (MSCs) exert a protective effect on tissues and stimulate tissue repair *in vitro* and *in vivo*, and MV transplantation is a novel cell-free therapeutic strategy for various degenerative disorders (Lee et al., 2012; Bian et al., 2014; Huang-Doran et al., 2017). In an *in vivo* experiment, Yang et al. (2019b) demonstrated for the first time that transplantation of HUMSCs-derived MVs could restore ovarian function in a POI mouse model by activating the PI3K-AKT signaling pathway and promoting damaged ovarian angiogenesis, in an *in vitro* experiment,HUMSCs-derived exosomes successfully rescued chemotherapy-induced apoptosis in rat ovarian granulosa cells (OGCs) (Sun et al., 2017). A recent study by Ding et al. (2020) demonstrated *in vitro* and *in vivo* that exosomes from HUMSCs rescued ovarian function in a POI mouse model, in which exosomes increased the proliferation rate and decreased the apoptosis rate of granulosa cells and ovaries in a dose-dependent manner in POI mice. To further investigate the effect of MVs derived from HUMSCs on the recovery of fertility in POI mice, *in vitro* fertilization and mating tests were performed and their progeny were evaluated, and the results showed that after treatment with MVs, the ovarian function of chemotherapy drug-induced POI mice was restored, the time to conception was shortened, and fertility was improved (Liu et al., 2020), It was further found that HUMSCs-MVs injected into the ovarian capsule of aged female mice promoted follicle development after peritoneal transplantation by carrying functional miRNAs, such as miR-146a-5p or miR-21-5p, which in turn activated the oocyte phosphatidylinositol 3-kinase (PI3K)/mTOR signaling pathway, resulting in increased oocyte production and improved oocyte quality and restoration of reduced fertility (Yang et al., 2020). Its clinical implementation may open up new perspectives for the diagnosis and treatment of translational medicine.

#### Anti-apoptotic Effects of Human Umbilical Cord Mesenchymal Stem Cells

It was shown that HUMSCs inhibit apoptosis of granulocytes in animal models of POF (Zhang et al., 2016), mainly related to the secretion of anti-apoptotic factors and growth factors by HUMSCs. In an animal model of POF induced by multiple chemotherapeutic agents, chemotherapy-induced cell damage was mainly associated with elevated levels of pro-apoptotic protein (Bax) and anti-apoptotic B lymphoma-2 (Bcl-2) (Sun et al., 2017). Cysteine proteinase-3 (caspase-3), an executor of apoptosis, also plays an important role in this process (Brentnall et al., 2013). In the POF rat model, HUMSCs transplantation can downregulate FSHR and Caspase-3 levels and reduce granulosa cell apoptosis (Zheng et al., 2019), as well as regulate follicle formation and inhibit Caspase-3-induced apoptosis through direct triggering effects on ovarian epithelium and/or by regulating tissue expression of CK 8/18, TGF-β, and PCNA, thereby indirectly enriching the ovarian ecotone (Elfayomy et al., 2016; Zhang et al., 2016, 2021), which in turn restores hormonal secretion and folliculogenesis disorders in POF rats and directly restores ovarian function at the level of the hormonal regulatory axis (Song et al., 2016). Similarly, in a POF mouse model, HUMSCs transplantation inhibits granulosa cell apoptosis and follicular atresia by upregulating AMH and FSHR expression in granulosa cells (Mohamed et al., 2019), elevates sex hormone levels (Wang et al., 2013; Jalalie et al., 2021), improves ovarian tissue apoptosis to an overall extent, and improves ovarian function (Shen et al., 2020). Granulosa cells play an important role in oocyte development and maturation, and HUMSCs have a positive impact on reproductive aging by improving granulosa cell metabolism and apoptosis rates both *in vitro* and *in vivo*.

#### Anti-fibrotic Effects of Human Umbilical Cord Mesenchymal Stem Cells

Fibroblasts overproliferate and are deposited in the extracellular matrix (ECM) of the ovary, and when they exceed a certain range, ovarian fibrosis can form (Zhou et al., 2017). It has been shown that the degree of fibrosis is lowest in the ovaries of young animals during the growth period, increases significantly in the ovaries of middle-aged and older breeding animals, and is most pronounced in the ovarian stroma of older animals (Briley et al., 2016). Similarly, in the POF mouse model researchers observed severe fibrosis in the ovaries, which was significantly reduced after transplantation of HUMSCs, thus suggesting that HUMSCs may secrete factors with therapeutic potential to repair or reverse fibrosis in the organ (Yang et al., 2019a). In chemically induced ovarian dysfunction (CIOD) and POI rat models, the expression of fibrosis markers (α-SMA) and the production of collagen type I (Collagen I) and collagen type III (Collagen III) were significantly inhibited in rats after transplantation of HUMSCs (Cui et al., 2020); the maximum tensile stress and maximum strain of damaged ovaries were enhanced, and to some extent restored the morphology, elasticity and toughness of the damaged ovaries and resulted in some restoration of ovarian function (Pan et al., 2017). The development of ovarian fibrosis is regulated by a complex network system, with many cytokines including matrix metalloproteinases (MMPs), matrix metalloproteinase tissue inhibitors (TIMPs), transforming growth factor β1 (TGFβ1), and connective tissue growth factor (CTGF) involved in fiber formation (Zhou et al., 2017); hepatocyte growth factor (HGF), basic fibroblast growth factor (bFGF), and adrenomedullin (ADM) (Azoury et al., 2008), among others, are involved in anti-fibrosis. During the anti-fibrotic process, HUMSCs inhibit fibroblast proliferation and reduce some of the deposition in the ECM, thus ameliorating ovarian fibrosis. At present, the molecular mechanism of antifibrosis by HUMSCs remains to be further investigated.

#### Pro-angiogenic Effects of Human Umbilical Cord Mesenchymal Stem Cells

Angiogenesis is also important in the recovery of ovarian function, providing nutrients and oxygen to the damaged ovaries. Factors such as VEGF secreted by HUMSCs are associated with angiogenesis. Studies have shown that VEGF promotes the length, area, and number of branch points of blood vessels (Beilmann et al., 2004). In a rat model of chemotherapy-induced ovarian dysfunction (CIOD), the intervention of HUMSCs elevated serum E2 and VEGF levels and promoted angiogenesis (Pan et al., 2017). In a mouse model of POI (Primary Ovarian Insufficiency), HUMSCs transplantation increased the body weight and follicle number of POI mice, induced ovarian angiogenesis, and restored their estrous cycle. 10 h after HUMSCs transplantation, the expression of ovarian angiogenic factors (including VEGF, IGF, angiogenin) was significantly up-regulated in POI mice. The high expression of angiogenic factors may play an important role in the recovery of ovarian function (Yang et al., 2019b). In addition, HUMSCs have a positive effect on the angiogenesis of ovarian tissues in the POF mouse model, which can significantly promote ovarian angiogenesis after transplantation, and with a significant increase in the expression of CD31 (platelet-endothelial cell adhesion molecule) (Yang et al., 2019a). It has been reported that Bone marrow-derived mesenchymal stem cells (BMSCs) transplants combined with cytokine HGF have a more pronounced effect on angiogenesis than BMSCs transplants alone (Su et al., 2013). During angiogenesis, the cytokines HGF and VEGF promote angiogenesis by inducing different vascular structural patterns. VEGF increases the length, area, and number of branching points of the induced vessels, whereas HGF mediates only the growth of vessel area, leading to an increase in the diameter of the vessel structure. The combination of these two cytokines leads to an increase in vascular diameter (Beilmann et al., 2004). HUMSCs and BMSCs belong to the same category of human umbilical cord mesenchymal stem cells and have great functional similarity. Whether we can combine HGF gene co-transplantation to achieve better results in the future deserves our better expectation.

#### Anti-inflammatory and Immunomodulatory Effects of Human Umbilical Cord Mesenchymal Stem Cells

HUMSCs play an important role in the induction of immune tolerance and exhibit better immunomodulatory properties than MSCs isolated from adult tissues (Macholdová et al., 2019), and their anti-inflammatory and immunomodulatory effects may be another mechanism by which HUMSCs ameliorate ovarian damage. It has been found that there is a large infiltration of immune cells, including T lymphocytes (van Kasteren et al., 2000; Li et al., 2008), B lymphocytes and natural killer cells, in the ovarian tissue of prematurely failing mice (Chernyshov et al., 2001); a disturbed balance of Th1/Th2 cytokine expression in the endometrial T lymphocyte subpopulation has also been found in POF patients (Wang et al., 2018), where uterine natural killer (uNK) cells play an important role in the endometrial local immune response plays an important role in maintaining the Th1/Th2 cytokine balance (Djurisic et al., 2015). Recently, Lu et al. (2019) showed that HUMSCs transplantation reduced endometrial Th1/Th2 cytokine release, decreased uNK cell expression, and promoted recovery of ovarian function and increased endometrial tolerance in mice with autoimmune POF. In an immune-induced rat POF model, transplantation of HUMSCs elevated serum concentrations of estradiol (E2), progesterone (P4), and anti-Müllerian hormone (AMH) and promoted granulosa cell proliferation (Wang et al., 2020b). The anti-inflammatory and immunomodulatory properties of HUMSCs provide us with new ideas for the treatment of immune-related ovarian injury, but their clinical utility still needs further investigation.

#### Anti-oxidative Stress Effect of Human Umbilical Cord Mesenchymal Stem Cells

Reactive oxygen species (ROS) generated during metabolic activities induced by oxidative stress is one of the important influences that induce ovarian aging (Liu et al., 2018). AMP-activated protein kinase (AMPK) is a cellular energy level sensor that is activated by increasing AMP/ATP ratio during oxidative stress. activation of AMPK inhibits mammalian target of rapamycin protein (mTOR) signaling (Adachi et al., 2014) activating autophagy, which is caused by high ROS levels generated by oxidative stress, thus controlling cellular energy homeostasis (Mizushima and Komatsu, 2011; Germic et al., 2019). It was found that transplantation of HUMSCs restored ovarian function and attenuated apoptosis of theca-interstitial cells (TICs) in POI rats in *in vivo* experiments; *in vitro* experiments, HUMSCs reduced autophagy levels in TICs by decreasing oxidative stress and regulating AMPK/mTOR signaling pathway, thereby attenuating of apoptosis (Lu et al., 2020). In a mouse model of accelerated ovarian aging, transplantation of HUMSCs promoted high expression of antioxidant enzymes and significantly improved ovarian function (Zhang et al., 2016). In another study, co-culture of HUMSCs with mouse embryos improved embryo quality by scavenging ROS and resulted in a significant increase in the number of blastocyst cells (Moshkdanian et al., 2011). Although studies directly regulating ovarian aging function through the modulation of oxidative stress by HUMSCs have not been reported, the fact that HUMSCs can improve fertility by scavenging ROS makes us look forward to this study.

### Potential of Human Umbilical Cord Mesenchymal Stem Cells to Be Induced Into Germ Cells

To develop a way to obtain germ cells *in vitro* to directly address female reproductive aging, scientists have long attempted to replicate germ cell differentiation or gametogenesis *in vitro* (Conti and Giudice, 2008). Under appropriate conditions, embryonic stem cells, bone marrow stem cells, and even stem cells derived from fetal porcine skin and rat exocrine pancreas, can be induced to differentiate toward the germ cell lineage. Moreover, the induced cells resemble germ cells morphologically and, in some cases, functionally (Takabayashi et al., 2001; Hübner et al., 2003; Clark et al., 2004; Geijsen et al., 2004; Dyce et al., 2006; Lacham-Kaplan et al., 2006; Nayernia et al., 2006). Induction of germ cells from HUMSCs is possible due to their pluripotent properties between embryonic stem cells and adult stem cells. Huang et al. (2010) attempted to obtain primary germ cells (PGC) derived from HUMSCs by using a number of inducers, and the induced cells showed germ cell-specific markers such as Oct4 (POUF5), Ckit, CD49 (alpha6), Stella (DDPA3) and Vasa (DDX4). To further verify the feasibility of differentiation of HUMSCs into germ cells, Qiu et al. (2013) transplanted HUMSCs into the germinal tubules and kidney capsule of mice, respectively, and found that the transplanted cells differentiated into germ-like cells in the recipient germinal tubules and kidney capsule. In 2015, transforming growth factor (TGFα, β) and basic fibroblast growth factor (bFGF) in a co-culture model using placental cell supplements provided a suitable environment for the induction of HUMSCs into PGCs and expression of oocyte-like markers, opening a new benchmark for obtaining primary germ cells (PGCs) and accessing oocytes-like cells *in vitro* (Asgari et al., 2015). Also, the oogenesis visualization system established by constructing a lentiviral vector (pTRIP-Figla-EGFP-puro) under the control of Figla promoter provides a more intuitive way to study the development and differentiation of stem cell germ cells (Chu et al., 2015). To find the best candidate for germ cell induction, researchers compared the ability of human amniotic membrane, chorionic villus and umbilical cord MSCs to differentiate into female germ cells and found that chorionic mesenchymal stem cells (hCMSCs) have a greater potential to differentiate into female germ cells. In addition, human umbilical cord MSCs of either male or female origin had the same differentiation potential to female germ cells (Asgari et al., 2017). Although HUMSCs are induced to have the ability to differentiate into germ cells *in vitro*, recent theories and reports still tend to suggest that they are practically unchanged *in vivo* (Ding et al., 2017).

## Conclusion and Perspectives

Considering the low immunogenicity of HUMSCs and their easy expansion *in vitro* to obtain in large quantities, the use of HUMSCs as candidates for reproductive senescence transplantation is promising. In animal models, HUMSCs improve reproductive senescence through paracrine, anti-apoptotic, anti-fibrotic, angiogenic, anti-inflammatory immunomodulatory and anti-oxidative stress effects, and perform well in restoring ovarian morphology and improving ovarian reserve capacity; they have also shown greater potential in *in vitro* induction as germ cells. However, evidence and safety studies on the effects of HUMSCs on the restoration of reproductive aging are still lacking in current clinical trials, and many specific mechanisms need to be further investigated. Meanwhile, in practical clinical applications, individual ovarian transplantation requires high requirements for the number of transplanted HUMSCs and the purity of transplanted cells, and further optimization of transplantation methods is needed to make HUMSCs transplantation safer and more effective in clinical treatment, which also presents new challenges for us.

## Author Contributions

QM carried out literature search, data collection, and analysis, and wrote the manuscript. HM and XL revised the manuscript. WX took part in design and revised the manuscript. All authors read and approved the manuscript.

## Conflict of Interest

The authors declare that the research was conducted in the absence of any commercial or financial relationships that could be construed as a potential conflict of interest.

## References

[B1] Adachi Y. Kanbayashi Y. Harata I. Ubagai R. Takimoto T. Suzuki K. (2014). Petasin activates AMP-activated protein kinase and modulates glucose metabolism. *J. Nat. Prod.* 77 1262–1269. 10.1021/np400867m 24871354

[B2] Akbari H. Eftekhar Vaghefi S. H. Shahedi A. Habibzadeh V. Mirshekari T. R. Ganjizadegan A. (2017). Mesenchymal stem cell-conditioned medium modulates apoptotic and stress-related gene expression, ameliorates maturation and allows for the development of immature human oocytes after artificial activation. *Genes* 8:371. 10.3390/genes8120371 29292728 PMC5748689

[B3] Araújo A. B. Salton G. D. Furlan J. M. Schneider N. Angeli M. H. Laureano ÁM. (2017). Comparison of human mesenchymal stromal cells from four neonatal tissues: Amniotic membrane, chorionic membrane, placental decidua and umbilical cord. *Cytotherapy* 19 577–585. 10.1016/j.jcyt.2017.03.001 28343898

[B4] Asgari H. R. Akbari M. Abbasi M. Ai J. Korouji M. Aliakbari F. (2015). Human Wharton’s jelly-derived mesenchymal stem cells express oocyte developmental genes during co-culture with placental cells. *Iran. J. Basic Med. Sci.* 18 22–29. 25810872 PMC4366739

[B5] Asgari H. R. Akbari M. Yazdekhasti H. Rajabi Z. Navid S. Aliakbari F. (2017). Comparison of human amniotic, chorionic, and umbilical cord multipotent mesenchymal stem cells regarding their capacity for differentiation toward female germ cells. *Cell Reprog.* 19 44–53. 10.1089/cell.2016.0035 28112985

[B6] Auersperg N. Wong A. S. Choi K. C. Kang S. K. Leung P. C. (2001). Ovarian surface epithelium: biology, endocrinology, and pathology. *Endocr. Rev.* 22 255–288. 10.1210/edrv.22.2.0422 11294827

[B7] Azoury J. Lee K. W. Georget V. Rassinier P. Leader B. Verlhac M. H. (2008). Spindle positioning in mouse oocytes relies on a dynamic meshwork of actin filaments. *Curr. Biol.* 18 1514–1519. 10.1016/j.cub.2008.08.044 18848445

[B8] Beilmann M. Birk G. Lenter M. C. (2004). Human primary co-culture angiogenesis assay reveals additive stimulation and different angiogenic properties of VEGF and HGF. *Cytokine* 26 178–185. 10.1016/j.cyto.2004.03.003 15149635

[B9] Bian S. Zhang L. Duan L. Wang X. Min Y. Yu H. (2014). Extracellular vesicles derived from human bone marrow mesenchymal stem cells promote angiogenesis in a rat myocardial infarction model. *J. Mol. Med.* 92 387–397. 10.1007/s00109-013-1110-5 24337504

[B10] Bidarimath M. Khalaj K. Kridli R. T. Kan F. W. Koti M. Tayade C. (2017). Extracellular vesicle mediated intercellular communication at the porcine maternal-fetal interface: a new paradigm for conceptus-endometrial cross-talk. *Sci. Rep.* 7:40476. 10.1038/srep40476 28079186 PMC5228034

[B11] Bongso A. Fong C. Y. (2013). The therapeutic potential, challenges and future clinical directions of stem cells from the Wharton’s jelly of the human umbilical cord. *Stem Cell Rev. Rep.* 9 226–240. 10.1007/s12015-012-9418-z 23233233

[B12] Brentnall M. Rodriguez-Menocal L. De Guevara R. L. Cepero E. Boise L. H. (2013). Caspase-9, caspase-3 and caspase-7 have distinct roles during intrinsic apoptosis. *BMC Cell Biol.* 14:32. 10.1186/1471-2121-14-32 23834359 PMC3710246

[B13] Briley S. M. Jasti S. Mccracken J. M. Hornick J. E. Fegley B. Pritchard M. T. (2016). Reproductive age-associated fibrosis in the stroma of the mammalian ovary. *Reproduction* 152 245–260. 10.1530/rep-16-0129 27491879 PMC4979755

[B14] Broekmans F. J. Soules M. R. Fauser B. C. (2009). Ovarian aging: mechanisms and clinical consequences. *Endocr. Rev.* 30 465–493. 10.1210/er.2009-0006 19589949

[B15] Brotherston J. (2015). Contraception meets HRT: seeking optimal management of the perimenopause. *Br. J. Gen. Pract.* 65 e630–e632. 10.3399/bjgp15X686689 26324501 PMC4540404

[B16] Chang H. M. Qiao J. Leung P. C. (2016). Oocyte-somatic cell interactions in the human ovary-novel role of bone morphogenetic proteins and growth differentiation factors. *Hum. Reprod. Update* 23 1–18. 10.1093/humupd/dmw039 27797914 PMC5155571

[B17] Chen G. Yue A. Ruan Z. Yin Y. Wang R. Ren Y. (2015). Comparison of biological characteristics of mesenchymal stem cells derived from maternal-origin placenta and Wharton’s jelly. *Stem Cell Res. The.* 6 228. 10.1186/s13287-015-0219-6 26607396 PMC4660673

[B18] Chen Q. Ke H. Luo X. Wang L. Wu Y. Tang S. (2020). Rare deleterious BUB1B variants induce premature ovarian insufficiency and early menopause. *Hum. Mol. Genet.* 29 2698–2707. 10.1093/hmg/ddaa153 32716490

[B19] Chernyshov V. P. Radysh T. V. Gura I. V. Tatarchuk T. P. Khominskaya Z. B. (2001). Immune disorders in women with premature ovarian failure in initial period. *Am. J. Reprod. Immunol.* 46 220–225. 10.1034/j.1600-0897.2001.d01-5.x 11554695

[B20] Chu Z. Niu B. Li N. Hu Y. Li J. Yu P. (2015). A lentiviral vector visualizing the germ cell specification in vitro under the control of Figla promoter. *Appl. Biochem. Biotechnol.* 176 66–75. 10.1007/s12010-015-1523-4 25652828

[B21] Clark A. T. Bodnar M. S. Fox M. Rodriquez R. T. Abeyta M. J. Firpo M. T. (2004). Spontaneous differentiation of germ cells from human embryonic stem cells in vitro. *Hum. Mol. Genet.* 13 727–739. 10.1093/hmg/ddh088 14962983

[B22] Conti M. Giudice L. (2008). From stem cells to germ cells and back again. *Nat. Med.* 14 1188–1190. 10.1038/nm1108-1188 18989302

[B23] Croitoru-Lamoury J. Lamoury F. M. J. Zaunders J. J. Veas L. A. Brew B. J. (2007). Human mesenchymal stem cells constitutively express chemokines and chemokine receptors that can be upregulated by cytokines, IFN-beta, and Copaxone. *J. Interferon Cytokine Res.* 27 53–64. 10.1089/jir.2006.0037 17266444

[B24] Cui L. Bao H. Liu Z. Man X. Liu H. Hou Y. (2020). hUMSCs regulate the differentiation of ovarian stromal cells via TGF-β(1)/Smad3 signaling pathway to inhibit ovarian fibrosis to repair ovarian function in POI rats. *Stem Cell Res. Ther.* 11:386. 10.1186/s13287-020-01904-3 32894203 PMC7487655

[B25] Ding C. Li H. Wang Y. Wang F. Wu H. Chen R. (2017). Different therapeutic effects of cells derived from human amniotic membrane on premature ovarian aging depend on distinct cellular biological characteristics. *Stem Cell Res. Ther.* 8:173. 10.1186/s13287-017-0613-3 28750654 PMC5530953

[B26] Ding C. Zhu L. Shen H. Lu J. Zou Q. Huang C. (2020). Exosomal miRNA-17-5p derived from human umbilical cord mesenchymal stem cells improves ovarian function in premature ovarian insufficiency by regulating SIRT7. *Stem Cells* 38 1137–1148. 10.1002/stem.3204 32442343

[B27] Ding L. Yan G. Wang B. Xu L. Gu Y. Ru T. (2018). Transplantation of UC-MSCs on collagen scaffold activates follicles in dormant ovaries of POF patients with long history of infertility. *Sci. China Life Sci.* 61 1554–1565. 10.1007/s11427-017-9272-2 29546669

[B28] Djurisic S. Skibsted L. Hviid T. V. (2015). A phenotypic analysis of regulatory T cells and uterine NK cells from first trimester pregnancies and associations with HLA-G. *Am. J. Reprod. Immunol.* 74 427–444. 10.1111/aji.12421 26293482

[B29] Donnez J. Dolmans M. M. (2018). Fertility preservation in women. *N. Engl. J. Med.* 378 400–401. 10.1056/NEJMc1715731 29365297

[B30] Dyce P. W. Wen L. Li J. (2006). In vitro germline potential of stem cells derived from fetal porcine skin. *Nat. Cell Biol.* 8 384–390. 10.1038/ncb1388 16565707

[B31] Elfayomy A. K. Almasry S. M. El-Tarhouny S. A. Eldomiaty M. A. (2016). Human umbilical cord blood-mesenchymal stem cells transplantation renovates the ovarian surface epithelium in a rat model of premature ovarian failure: possible direct and indirect effects. *Tissue Cell* 48 370–382. 10.1016/j.tice.2016.05.001 27233913

[B32] Faddy M. J. Gosden R. G. Gougeon A. Richardson S. J. Nelson J. F. (1992). Accelerated disappearance of ovarian follicles in mid-life: implications for forecasting menopause. *Hum. Reprod.* 7 1342–1346. 10.1093/oxfordjournals.humrep.a137570 1291557

[B33] Fong C. Y. Chak L. L. Biswas A. Tan J. H. Gauthaman K. Chan W. K. (2011). Human Wharton’s jelly stem cells have unique transcriptome profiles compared to human embryonic stem cells and other mesenchymal stem cells. *Stem Cell Rev. Rep.* 7 1–16. 10.1007/s12015-010-9166-x 20602182

[B34] Fu X. He Y. Xie C. Liu W. (2008). Bone marrow mesenchymal stem cell transplantation improves ovarian function and structure in rats with chemotherapy-induced ovarian damage. *Cytotherapy* 10 353–363. 10.1080/14653240802035926 18574768

[B35] Geijsen N. Horoschak M. Kim K. Gribnau J. Eggan K. Daley G. Q. (2004). Derivation of embryonic germ cells and male gametes from embryonic stem cells. *Nature* 427 148–154. 10.1038/nature02247 14668819

[B36] Germic N. Frangez Z. Yousefi S. Simon H. U. (2019). Regulation of the innate immune system by autophagy: monocytes, macrophages, dendritic cells and antigen presentation. *Cell Death Differ.* 26 715–727. 10.1038/s41418-019-0297-6 30737475 PMC6460400

[B37] Guan Y. T. Xie Y. Li D. S. Zhu Y. Y. Zhang X. L. Feng Y. L. (2019). Comparison of biological characteristics of mesenchymal stem cells derived from the human umbilical cord and decidua parietalis. *Mol. Med. Rep.* 20 633–639. 10.3892/mmr.2019.10286 31180542 PMC6579987

[B38] György B. Szabó T. G. Pásztói M. Pál Z. Misják P. Aradi B. (2011). Membrane vesicles, current state-of-the-art: emerging role of extracellular vesicles. *Cell. Mol. Life Sci.* 68 2667–2688. 10.1007/s00018-011-0689-3 21560073 PMC3142546

[B39] Hong L. Yan L. Xin Z. Hao J. Liu W. Wang S. (2020). Protective effects of human umbilical cord mesenchymal stem cell-derived conditioned medium on ovarian damage. *J. Mol. Cell. Biol.* 12 372–385. 10.1093/jmcb/mjz105 31742349 PMC7288746

[B40] Huang P. Lin L. M. Wu X. Y. Tang Q. L. Feng X. Y. Lin G. Y. (2010). Differentiation of human umbilical cord wharton’s jelly-derived mesenchymal stem cells into germ-like cells in vitro. *J. Cell. Biochem.* 109 747–754. 10.1002/jcb.22453 20052672

[B41] Huang-Doran I. Zhang C. Y. Vidal-Puig A. (2017). Extracellular vesicles: novel mediators of cell communication in metabolic disease. *Trends Endocrinol. Metab.* 28 3–18. 10.1016/j.tem.2016.10.003 27810172

[B42] Hübner K. Fuhrmann G. Christenson L. K. Kehler J. Reinbold R. De La Fuente R. (2003). Derivation of oocytes from mouse embryonic stem cells. *Science* 300 1251–1256. 10.1126/science.1083452 12730498

[B43] Jalalie L. Rezaee M. A. Rezaie M. J. Jalili A. Raoofi A. Rustamzade A. (2021). Human umbilical cord mesenchymal stem cells improve morphometric and histopathologic changes of cyclophosphamide-injured ovarian follicles in mouse model of premature ovarian failure. *Acta Histochem.* 123:151658. 10.1016/j.acthis.2020.151658 33249312

[B44] Jia Y. Shi X. Xie Y. Xie X. Wang Y. Li S. (2017). Human umbilical cord stem cell conditioned medium versus serum-free culture medium in the treatment of cryopreserved human ovarian tissues in in-vitro culture: a randomized controlled trial. *Stem Cell Res. Ther.* 8:152. 10.1186/s13287-017-0604-4 28646900 PMC5482969

[B45] Lacham-Kaplan O. Chy H. Trounson A. (2006). Testicular cell conditioned medium supports differentiation of embryonic stem cells into ovarian structures containing oocytes. *Stem Cells* 24 266–273. 10.1634/stemcells.2005-0204 16109761

[B46] Lee C. Mitsialis S. A. Aslam M. Vitali S. H. Vergadi E. Konstantinou G. (2012). Exosomes mediate the cytoprotective action of mesenchymal stromal cells on hypoxia-induced pulmonary hypertension. *Circulation* 126 2601–2611. 10.1161/circulationaha.112.114173 23114789 PMC3979353

[B47] Li J. Jin H. Zhang F. Du X. Zhao G. Yu Y. (2008). Treatment of autoimmune ovarian disease by co-administration with mouse zona pellucida protein 3 and DNA vaccine through induction of adaptive regulatory T cells. *J. Gene. Med.* 10 810–820. 10.1002/jgm.1200 18452236

[B48] Li J. Mao Q. He J. She H. Zhang Z. Yin C. (2017). Human umbilical cord mesenchymal stem cells improve the reserve function of perimenopausal ovary via a paracrine mechanism. *Stem Cell Res. Ther.* 8:55. 10.1186/s13287-017-0514-5 28279229 PMC5345137

[B49] Liu C. Yin H. Jiang H. Du X. Wang C. Liu Y. (2020). Extracellular vesicles derived from mesenchymal stem cells recover fertility of premature ovarian insufficiency mice and the effects on their offspring. *Cell Transplant.* 29:963689720923575. 10.1177/0963689720923575 32363925 PMC7586265

[B50] Liu J. Zhang H. Zhang Y. Li N. Wen Y. Cao F. (2014). Homing and restorative effects of bone marrow-derived mesenchymal stem cells on cisplatin injured ovaries in rats. *Mol. Cells* 37 865–872. 10.14348/molcells.2014.0145 25410907 PMC4275703

[B51] Liu X. Lin X. Zhang S. Guo C. Li J. Mi Y. (2018). Lycopene ameliorates oxidative stress in the aging chicken ovary via activation of Nrf2/HO-1 pathway. *Aging* 10 2016–2036. 10.18632/aging.101526 30115814 PMC6128425

[B52] Lu X. Bao H. Cui L. Zhu W. Zhang L. Xu Z. (2020). hUMSC transplantation restores ovarian function in POI rats by inhibiting autophagy of theca-interstitial cells via the AMPK/mTOR signaling pathway. *Stem Cell Res. Ther.* 11:268. 10.1186/s13287-020-01784-7 32620136 PMC7333437

[B53] Lu X. Cui J. Cui L. Luo Q. Cao Q. Yuan W. (2019). The effects of human umbilical cord-derived mesenchymal stem cell transplantation on endometrial receptivity are associated with Th1/Th2 balance change and uNK cell expression of uterine in autoimmune premature ovarian failure mice. *Stem Cell Res. Ther.* 10:214. 10.1186/s13287-019-1313-y 31331391 PMC6647296

[B54] Macholdová K. Macháčková E. Prošková V. Hromadníková I. Klubal R. (2019). Latest findings on the placenta from the point of view of immunology, tolerance and mesenchymal stem cells. *Ceska Gynekol* 84 154–160. 31238687

[B55] May-Panloup P. Boucret L. Chao De La Barca J. M. Desquiret-Dumas V. Ferré-L’hotellier V. Moriničre C. (2016). Ovarian ageing: the role of mitochondria in oocytes and follicles. *Hum. Reprod. Update* 22 725–743. 10.1093/humupd/dmw028 27562289

[B56] Mizushima N. Komatsu M. (2011). Autophagy: renovation of cells and tissues. *Cell* 147 728–741. 10.1016/j.cell.2011.10.026 22078875

[B57] Mohamed S. A. Shalaby S. Brakta S. Elam L. Elsharoud A. Al-Hendy A. (2019). Umbilical cord blood mesenchymal stem cells as an infertility treatment for chemotherapy induced premature ovarian insufficiency. *Biomedicines* 7:7. 10.3390/biomedicines7010007 30669278 PMC6466426

[B58] Moshkdanian G. Nematollahi-Mahani S. N. Pouya F. Nematollahi-Mahani A. (2011). Antioxidants rescue stressed embryos at a rate comparable with co-culturing of embryos with human umbilical cord mesenchymal cells. *J. Assist. Reprod. Genet.* 28 343–349. 10.1007/s10815-010-9529-x 21207131 PMC3114960

[B59] Nayernia K. Lee J. H. Drusenheimer N. Nolte J. Wulf G. Dressel R. (2006). Derivation of male germ cells from bone marrow stem cells. *Lab. Invest.* 86 654–663. 10.1038/labinvest.3700429 16652109

[B60] Ortmann O. Dören M. Windler E. (2011). Hormone therapy in perimenopause and postmenopause (HT). Interdisciplinary S3 guideline, association of the scientific medical societies in germany AWMF 015/062-short version. *Arch. Gynecol. Obstet.* 284 343–355. 10.1007/s00404-011-1878-x 21431845 PMC3133644

[B61] Pan Y. Zhang L. Zhang X. Hu C. Liu R. (2017). Biological and biomechanical analysis of two types of mesenchymal stem cells for intervention in chemotherapy-induced ovarian dysfunction. *Arch. Gynecol. Obstet.* 295 247–252. 10.1007/s00404-016-4224-5 27928675

[B62] Persani L. Rossetti R. Cacciatore C. (2010). Genes involved in human premature ovarian failure. *J. Mol. Endocrinol.* 45 257–279. 10.1677/jme-10-0070 20668067

[B63] Qiu P. Bai Y. Pan S. Li W. Liu W. Hua J. (2013). Gender depended potentiality of differentiation of human umbilical cord mesenchymal stem cells into oocyte-Like cells in vitro. *Cell. Biochem. Funct.* 31 365–373. 10.1002/cbf.2981 23657870

[B64] Rees M. (2011). Management of the menopause: integrated health-care pathway for the menopausal woman. *Menopause Int.* 17 50–54. 10.1258/mi.2011.011013 21693499

[B65] Richardson S. J. Senikas V. Nelson J. F. (1987). Follicular depletion during the menopausal transition: evidence for accelerated loss and ultimate exhaustion. *J. Clin. Endocrinol. Metab.* 65 1231–1237. 10.1210/jcem-65-6-1231 3119654

[B66] Seshareddy K. Troyer D. Weiss M. L. (2008). Method to isolate mesenchymal-like cells from Wharton’s Jelly of umbilical cord. *Methods Cell Biol.* 86 101–119. 10.1016/s0091-679x(08)00006-x 18442646

[B67] Shen J. Cao D. Sun J. L. (2020). Ability of human umbilical cord mesenchymal stem cells to repair chemotherapy-induced premature ovarian failure. *World J. Stem Cells* 12 277–287. 10.4252/wjsc.v12.i4.277 32399136 PMC7202924

[B68] Song D. Zhong Y. Qian C. Zou Q. Ou J. Shi Y. (2016). Human umbilical cord mesenchymal stem cells therapy in cyclophosphamide-induced premature ovarian failure rat model. *Biomed. Res. Int.* 2016:2517514. 10.1155/2016/2517514 27047962 PMC4800076

[B69] Su G. H. Sun Y. F. Lu Y. X. Shuai X. X. Liao Y. H. Liu Q. Y. (2013). Hepatocyte growth factor gene-modified bone marrow-derived mesenchymal stem cells transplantation promotes angiogenesis in a rat model of hindlimb ischemia. *J. Huazhong Univ. Sci. Technol. Med. Sci.* 33 511–519. 10.1007/s11596-013-1151-6 23904370

[B70] Sun L. Li D. Song K. Wei J. Yao S. Li Z. (2017). Exosomes derived from human umbilical cord mesenchymal stem cells protect against cisplatin-induced ovarian granulosa cell stress and apoptosis in vitro. *Sci. Rep.* 7:2552. 10.1038/s41598-017-02786-x 28566720 PMC5451424

[B71] Takabayashi S. Sasaoka Y. Yamashita M. Tokumoto T. Ishikawa K. Noguchi M. (2001). Novel growth factor supporting survival of murine primordial germ cells: evidence from conditioned medium of ter fetal gonadal somatic cells. *Mol. Reprod. Dev.* 60 384–396. 10.1002/mrd.1101 11599050

[B72] van Kasteren Y. M. Von Blomberg M. Hoek A. De Koning C. Lambalk N. Van Montfrans J. (2000). Incipient ovarian failure and premature ovarian failure show the same immunological profile. *Am. J. Reprod. Immunol.* 43 359–366. 10.1111/j.8755-8920.2000.430605.x 10910195

[B73] Wang L. Yu C. Chang T. Zhang M. Song S. Xiong C. (2020a). In situ repair abilities of human umbilical cord-derived mesenchymal stem cells and autocrosslinked hyaluronic acid gel complex in rhesus monkeys with intrauterine adhesion. *Sci. Adv.* 6:eaba6357. 10.1126/sciadv.aba6357 32494750 PMC7244313

[B74] Wang Z. Wei Q. Wang H. Han L. Dai H. Qian X. (2020b). Mesenchymal stem cell therapy using human umbilical cord in a rat model of autoimmune-induced premature ovarian failure. *Stem Cells Int.* 2020:3249495. 10.1155/2020/3249495 32714395 PMC7355366

[B75] Wang P. Lu Y. Chen S. Chen Y. Hu C. Zuo Y. (2018). Protective function of Bu Shen Huo Xue formula on the immunity of B6AF1 mice with experimental autoimmune premature ovarian failure. *Exp. Ther. Med.* 15 3302–3310. 10.3892/etm.2018.5804 29545848 PMC5840928

[B76] Wang S. Yu L. Sun M. Mu S. Wang C. Wang D. (2013). The therapeutic potential of umbilical cord mesenchymal stem cells in mice premature ovarian failure. *Biomed. Res. Int.* 2013:690491. 10.1155/2013/690491 23998127 PMC3753743

[B77] Weber M. T. Maki P. M. Mcdermott M. P. (2014). Cognition and mood in perimenopause: a systematic review and meta-analysis. *J. Steroid Biochem. Mol. Biol.* 142 90–98. 10.1016/j.jsbmb.2013.06.001 23770320 PMC3830624

[B78] Wu M. Zhang R. Zou Q. Chen Y. Zhou M. Li X. (2018). Comparison of the biological characteristics of mesenchymal stem cells derived from the human placenta and umbilical cord. *Sci. Rep.* 8:5014. 10.1038/s41598-018-23396-1 29568084 PMC5864926

[B79] Wu Z. Zhang S. Zhou L. Cai J. Tan J. Gao X. (2017). Thromboembolism induced by umbilical cord mesenchymal stem cell infusion: a report of two cases and literature review. *Transplant. Proc.* 49 1656–1658. 10.1016/j.transproceed.2017.03.078 28838459

[B80] Xu L. Ding L. Wang L. Cao Y. Zhu H. Lu J. (2017). Umbilical cord-derived mesenchymal stem cells on scaffolds facilitate collagen degradation via upregulation of MMP-9 in rat uterine scars. *Stem Cell Res. Ther.* 8:84. 10.1186/s13287-017-0535-0 28420433 PMC5395893

[B81] Yan L. Wu Y. Li L. Wu J. Zhao F. Gao Z. (2020). Clinical analysis of human umbilical cord mesenchymal stem cell allotransplantation in patients with premature ovarian insufficiency. *Cell Prolif.* 53:e12938. 10.1111/cpr.12938 33124125 PMC7705906

[B82] Yang W. Zhang J. Xu B. He Y. Liu W. Li J. (2020). HucMSC-derived exosomes mitigate the age-related retardation of fertility in female mice. *Mol. Ther.* 28 1200–1213. 10.1016/j.ymthe.2020.02.003 32097602 PMC7132622

[B83] Yang Y. Lei L. Wang S. Sheng X. Yan G. Xu L. (2019a). Transplantation of umbilical cord-derived mesenchymal stem cells on a collagen scaffold improves ovarian function in a premature ovarian failure model of mice. *Vitro Cell Dev. Biol. Anim.* 55 302–311. 10.1007/s11626-019-00337-4 30820812

[B84] Yang Z. Du X. Wang C. Zhang J. Liu C. Li Y. (2019b). Therapeutic effects of human umbilical cord mesenchymal stem cell-derived microvesicles on premature ovarian insufficiency in mice. *Stem Cell Res. Ther.* 10:250. 10.1186/s13287-019-1327-5 31412919 PMC6693188

[B85] Zhang J. Xiong J. Fang L. Lu Z. Wu M. Shi L. (2016). The protective effects of human umbilical cord mesenchymal stem cells on damaged ovarian function: a comparative study. *Biosci. Trends* 10 265–276. 10.5582/bst.2016.01125 27464625

[B86] Zhang X. Zhang L. Li Y. Yin Z. Feng Y. Ji Y. (2021). Human umbilical cord mesenchymal stem cells (hUCMSCs) promotes the recovery of ovarian function in a rat model of premature ovarian failure (POF). *Gynecol. Endocrinol.* 2021 1–5. 10.1080/09513590.2021.1878133 33491494

[B87] Zhao Y. X. Chen S. R. Su P. P. Huang F. H. Shi Y. C. Shi Q. Y. (2019). Using mesenchymal stem cells to treat female infertility: an update on female reproductive diseases. *Stem Cells Int.* 2019:9071720. 10.1155/2019/9071720 31885630 PMC6925937

[B88] Zheng Q. Fu X. Jiang J. Zhang N. Zou L. Wang W. (2019). Umbilical cord mesenchymal stem cell transplantation prevents chemotherapy-induced ovarian failure via the NGF/TrkA pathway in rats. *Biomed. Res. Int.* 2019:6539294. 10.1155/2019/6539294 31240219 PMC6556346

[B89] Zhou F. Shi L. B. Zhang S. Y. (2017). Ovarian fibrosis: a phenomenon of concern. *Chin. Med. J.* 130 365–371. 10.4103/0366-6999.198931 28139522 PMC5308021

[B90] Zhu S.-F. Hu H.-B. Xu H.-Y. Fu X.-F. Peng D.-X. Su W.-Y. (2015). Human umbilical cord mesenchymal stem cell transplantation restores damaged ovaries. *J. Cell. Mol. Med.* 19 2108–2117. 10.1111/jcmm.12571 25922900 PMC4568915

